# Trichlorido(1,3-dimethyl-2,3-di­hydro-1*H*-imidazol-2-yl­idene-κ*C* ^2^)aluminium(III)

**DOI:** 10.1107/S1600536813018254

**Published:** 2013-07-06

**Authors:** Chong Tian, Xiaofei Gao, Qiao Chen, Wanli Nie, Maxim V. Borzov

**Affiliations:** a College of Chemistry, Leshan Normal University, Binhe Rd 778, Leshan 614000, Sichuan Province, People’s Republic of China; b Zhengzhou Research Institute of Comprehensive Utilization of Mineral Resourses of CAGS, Longhai Rd 328, Zhengzhou 450006, Henan Province, People’s Republic of China

## Abstract

The title compound, [Al(C_5_H_8_N_2_)Cl_3_], was prepared by a thermolytic decomposition under high-vacuum conditions and presents a formal adduct of an Arduengo carbene, 1,3-dimethyl-1*H*-imidazol-2-yl­idene, and aluminium trichloride. The Al atom adopts a pseudo-tetra­hedral CCl_3_ coordination environment. All N and C atoms, the Al atom, one of the Cl atoms, and all aromatic H atoms of the mol­ecule lie on a mirror plane. As a result of the mirror symmetry of the mol­ecule, the H atoms of all methyl groups are disordered between symmetry-equivalent positions.

## Related literature

For related structurally characterized Arduengo carbene Al*X*_3_ (*X* = Cl, I) adducts, see: Stasch *et al.* (2004 ▶); Ghadwal *et al.* (2009 ▶); Bantu *et al.* (2009 ▶). For thermolytic inter­conversion of sterically non-hindered 1,3-dialkyl-1*H*-imidazolium salts with BF_4_^−^ and PF_6_^−^ anions into Arduengo carbene adducts with BF_3_ and PF_5_, see: Tian *et al.* (2012 ▶). For the crystal structure of the precursor employed in the synthesis of the title compound, see: Tian *et al.* (2013 ▶). For a description of the Cambridge Structural Database, see: Allen (2002 ▶).
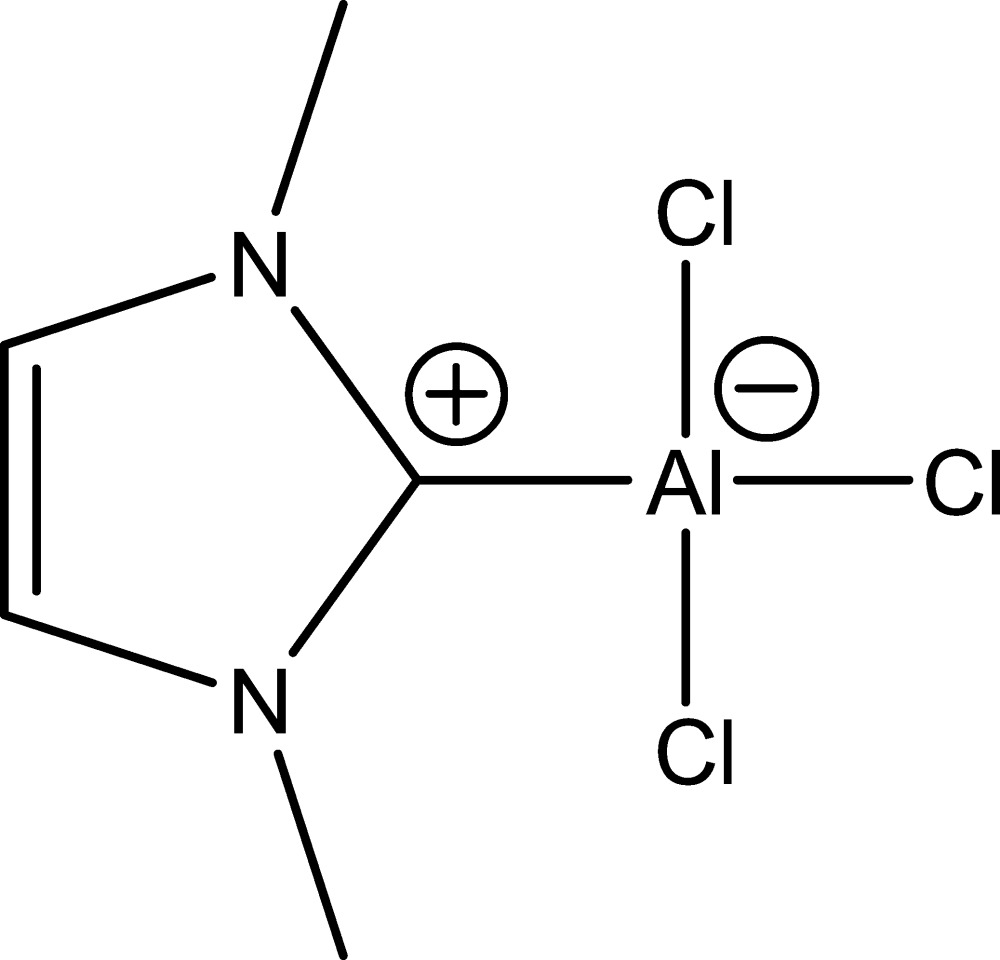


## Experimental

### 

#### Crystal data

[Al(C_5_H_8_N_2_)Cl_3_]
*M* *_r_* = 229.46
Orthorhombic, 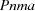

*a* = 8.9075 (7) Å
*b* = 7.3903 (6) Å
*c* = 15.3253 (12) Å
*V* = 1008.85 (14) Å^3^
*Z* = 4
Mo *K*α radiation
μ = 0.94 mm^−1^
*T* = 296 K
0.40 × 0.38 × 0.20 mm

#### Data collection

Bruker SMART APEXII diffractometer
Absorption correction: multi-scan (*SADABS*; Bruker, 2007 ▶) *T*_min_ = 0.706, *T*_max_ = 0.835
5094 measured reflections
1062 independent reflections
968 reflections with *I* > 2σ(*I*)
*R*_int_ = 0.022

#### Refinement

*R*[*F* ^2^ > 2σ(*F* ^2^)] = 0.033
*wR*(*F* ^2^) = 0.087
*S* = 1.07
1062 reflections
66 parameters
H-atom parameters constrained
Δρ_max_ = 0.40 e Å^−3^
Δρ_min_ = −0.43 e Å^−3^

### 

Data collection: *APEX2* (Bruker, 2007 ▶); cell refinement: *SAINT* (Bruker, 2007 ▶); data reduction: *SAINT*; program(s) used to solve structure: *SHELXS97* (Sheldrick, 2008 ▶); program(s) used to refine structure: *SHELXL97* (Sheldrick, 2008 ▶); molecular graphics: *SHELXTL* (Sheldrick, 2008 ▶) and *OLEX2* (Dolomanov *et al.*, 2009 ▶); software used to prepare material for publication: *SHELXTL* and *OLEX2*.

## Supplementary Material

Crystal structure: contains datablock(s) I, global. DOI: 10.1107/S1600536813018254/wm2754sup1.cif

Structure factors: contains datablock(s) I. DOI: 10.1107/S1600536813018254/wm2754Isup2.hkl

Supplementary material file. DOI: 10.1107/S1600536813018254/wm2754Isup3.cdx

Additional supplementary materials:  crystallographic information; 3D view; checkCIF report

## Figures and Tables

**Table 1:** Selected bond lengths (Å)

Cl1—Al1	2.1193 (12)
Cl2—Al1	2.1290 (7)
Al1—C1	2.006 (3)
Al1—Cl2^i^	2.1291 (7)

Symmetry code: (i) 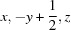.

**Fig. 1.**
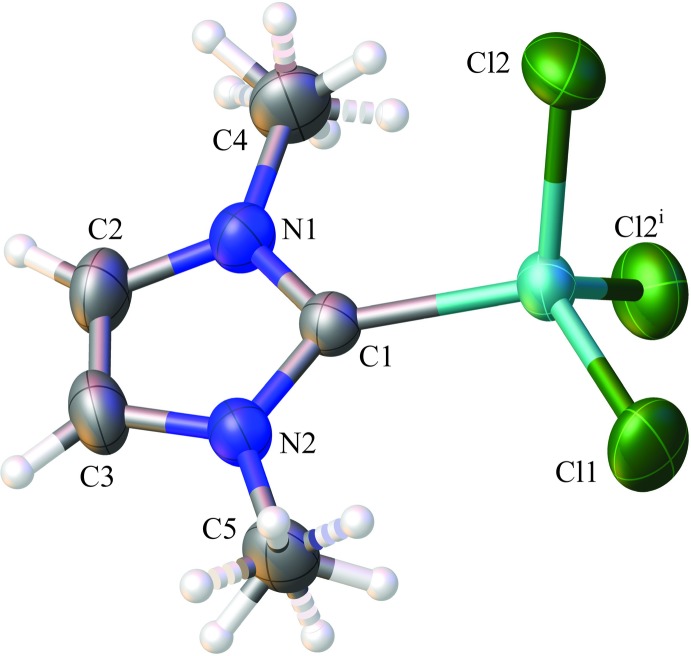
The molecular structure of compound (I). Thermal displacement ellipsoids are shown at the 50% probability level. Bonds to symmetry-equivalent disordered H-atoms are dashed. [Symmetry code: (i) x, -y + 1/2, z.]

## supplementary crystallographic information

### Comment

Structurally characterized Al*X*_3_ (*X* = Cl, I) adducts with Arduengo carbenes (ACs) are known since 2004 (Stasch *et al.*, 2004) and are still few (Ghadwal *et al.*, 2009; Bantu *et al.*, 2009). Except of the first described representative, *viz*. trichlorido(1,3,4,5-dimethyl-2,3-dihydro-1*H*-imidazol-2-ylidene-κ*C*^2^)aluminium (Stasch *et al.*, 2004), they all present complexes with sterically hindered ACs which bear either mesityl or 2,6-diisopropylphenyl substituents at the N-atoms. Of interest, in all four structurally characterized *X*_3_Al—AC adducts, the Al atoms are in a tetrahedral coordination environment as shown by an analysis of the structures compiled in the Cambridge Structural Database (Allen, 2002) [CSD; Version 5.34, release May 2013; 4 entries, 4 fragments].

Preparation of all Al complexes mentioned above includes, as a step, generation of a free AC by deprotonation of a corresponding imidazolium salt with a strong base under mild conditions. This seriously limits the method due to the known thermal instability of sterically non-hindered ACs even in solution. Recently, we developed a facile route to BF_3_ and PF_5_ adducts with sterically non-hindered ACs by a thermolytic decomposition of related imidazolium salts with [BF_4_^-^] and/or [PF_5_^-^] anions under high-vacuum conditions [573–673 K, 1.3–2.0×10 ^-1^ Pa; Tian *et al.* (2012)]. Heating of an equimolar mixture of 1,3-dimethyl-1*H*-imidazolium (hydrogen difluoride), [C_5_H_8_N_2_^+^][HF_2_^-^], (II), and AlCl_3_ under the same conditions followed by re-crystallization precedures led to formation of the title compound (I), C_5_H_8_N_2_AlCl_3_, in a moderate yield (see Experimental for the details; for the crystal structure of (II), see: Tian *et al.*, 2013).

Compound (I) presents a formal adduct of an Arduengo carbene, 1,3-dimethyl-1*H*-imidazol-2-ylidene, and aluminium trichloride. The Al-atom adopts a pseudo-tetrahedral coordination environment, defined by the three Cl atoms and the carbene C atom (Table 1). All N- and C-atoms, the Al-atom, one of the Cl-atoms, and all aromatic H-atoms of the molecule lie on a mirror plane at (*x*, 1/4, *z*). The H-atoms of methyl groups are disordered between symmetry equivalent positions (Fig. 1).

### Experimental

Acetonitrile and toluene solvents were kept over and distilled from CaH_2_ and/or Na/K alloy under Ar atmosphere, respectively. AlCl_3_ was sublimed in high vacuum prior to use. ^1^H NMR spectra were recorded on a Varian 400 INOVA instrument in CD_3_CN at 400 MHz and 298 K, with the signal of the residual solvent protons [δ(H) = 1.94 p.p.m.] used as an internal reference.

The imidazolium salt (II) was prepared by a reaction of AgF (1.27 g, 10.0 mmol) and 1,3-dimethyl-1*H*-imidazolium iodide (2.24 g, 10.0 mmol) in distilled water (25 ml). The precipitate of AgI was filtered off, the filtrate was concentrated till dryness, and dried on the high-vacuum line (1.3–2.0×10 ^-1^ Pa) at 323 K. Recrystallization from an ethanol/acetone mixture (1: 1) followed by double recrystallization from dry acetonitrile gave 0.93 g (6.8 mmol, 68%) of (II) as colorless crystals. ^1^H NMR δ p.p.m.: 3.85 (s, 6H, CH_3_), 7.44 (s, 2H, CH=CH), 9.45 (s, 1H, NCHN).

Compound (I): Crystalline (II) (0.93 g, 6.8 mmol) and AlCl_3_ (0.91 g, 6.8 mmol) were placed into a small apparatus for distillation of high-melting compounds, the system was connected to the high-vacuum line (1.3–2.0×10 ^-1^ Pa) through a liq. N_2_ cooled trap, evacuated, and heated. At approximately 423 K, the signs of melting were observed and then a strongly exothermic reaction accompanied by a gas evolution started. The temperature of the reaction mixture was then increased up to 573 K and the crude product was collected in the receiver as a reddish oil (reaction vessel temperature 573–673 K). The crude compound of (I) (1.03 g, 4.5 mmol, 66%) was allowed to crystallize in a refrigerator (270 K, 7 days). ^1^H NMR δ p.p.m.: 3.98 (s, 6H, CH_3_), 7.24 (s, 2H, CH=CH). Single crystal of (I) suitable for the X-ray diffraction analysis were grown by recrystallization of crude (I) from dry toluene, mounted in a Lindemann glass capillary (Ø 0.5 mm, glove box, N_2_ atmosphere) and sealed off.

### Refinement

The H atoms were treated as riding atoms with distances C—H = 0.96 (CH_3_), 0.93 Å (C_Ar_H) and *U*_iso_(H) = 1.5 *U*_eq_(C), 1.2 *U*_eq_(C), respectively. The H-atoms of the methyl groups are disordered due to the mirror symmetry of the aromatic ring; hance they were refined with half-occupancy.

### Crystal data

**Table:**

[Al(C_5_H_8_N_2_)Cl_3_]	*F*(000) = 464
*M**_r_* = 229.46	*D*_x_ = 1.511 Mg m^−^^3^
Orthorhombic, *P**n**m**a*	Mo *K*α radiation, λ = 0.71073 Å
Hall symbol: -P 2ac 2n	Cell parameters from 4672 reflections
*a* = 8.9075 (7) Å	θ = 2.7–28.3°
*b* = 7.3903 (6) Å	µ = 0.94 mm^−^^1^
*c* = 15.3253 (12) Å	*T* = 296 K
*V* = 1008.85 (14) Å^3^	Block, colourless
*Z* = 4	0.40 × 0.38 × 0.20 mm

### Data collection

**Table:**

Bruker SMART APEXII diffractometer	1062 independent reflections
Radiation source: fine-focus sealed tube	968 reflections with *I* > 2σ(*I*)
Graphite monochromator	*R*_int_ = 0.022
Detector resolution: 8.333 pixels mm^-1^	θ_max_ = 26.0°, θ_min_ = 2.6°
phi and ω scans	*h* = −7→10
Absorption correction: multi-scan (*SADABS*; Bruker, 2007)	*k* = −9→9
*T*_min_ = 0.706, *T*_max_ = 0.835	*l* = −18→17
5094 measured reflections	

### Refinement

**Table:**

Refinement on *F*^2^	Primary atom site location: structure-invariant direct methods
Least-squares matrix: full	Secondary atom site location: difference Fourier map
*R*[*F*^2^ > 2σ(*F*^2^)] = 0.033	Hydrogen site location: inferred from neighbouring sites
*wR*(*F*^2^) = 0.087	H-atom parameters constrained
*S* = 1.07	*w* = 1/[σ^2^(*F*_o_^2^) + (0.0365*P*)^2^ + 0.7006*P*] where *P* = (*F*_o_^2^ + 2*F*_c_^2^)/3
1062 reflections	(Δ/σ)_max_ < 0.001
66 parameters	Δρ_max_ = 0.40 e Å^−^^3^
0 restraints	Δρ_min_ = −0.43 e Å^−^^3^

### Special details

**Table:**

Geometry. All e.s.d.'s (except the e.s.d. in the dihedral angle between two l.s. planes) are estimated using the full covariance matrix. The cell e.s.d.'s are taken into account individually in the estimation of e.s.d.'s in distances, angles and torsion angles; correlations between e.s.d.'s in cell parameters are only used when they are defined by crystal symmetry. An approximate (isotropic) treatment of cell e.s.d.'s is used for estimating e.s.d.'s involving l.s. planes.
Refinement. Refinement of *F*^2^ against ALL reflections. The weighted *R*-factor *wR* and goodness of fit *S* are based on *F*^2^, conventional *R*-factors *R* are based on *F*, with *F* set to zero for negative *F*^2^. The threshold expression of *F*^2^ > σ(*F*^2^) is used only for calculating *R*-factors(gt) *etc*. and is not relevant to the choice of reflections for refinement. *R*-factors based on *F*^2^ are statistically about twice as large as those based on *F*, and *R*- factors based on ALL data will be even larger.

### Fractional atomic coordinates and isotropic or equivalent isotropic displacement parameters (Å^2^)

**Table:**

	*x*	*y*	*z*	*U*_iso_*/*U*_eq_	Occ. (<1)
Cl1	−0.07057 (9)	0.2500	0.44325 (6)	0.0726 (3)	
Cl2	0.18333 (7)	0.01243 (9)	0.31551 (4)	0.0633 (2)	
Al1	0.15020 (9)	0.2500	0.39170 (5)	0.0399 (2)	
N1	0.4590 (3)	0.2500	0.46678 (15)	0.0428 (5)	
N2	0.3009 (3)	0.2500	0.57221 (14)	0.0405 (5)	
C1	0.3100 (3)	0.2500	0.48390 (17)	0.0362 (6)	
C2	0.5401 (3)	0.2500	0.5426 (2)	0.0546 (8)	
H2	0.6441	0.2500	0.5474	0.066*	
C3	0.4420 (4)	0.2500	0.6082 (2)	0.0537 (8)	
H3	0.4649	0.2500	0.6675	0.064*	
C4	0.5262 (4)	0.2500	0.3794 (2)	0.0589 (8)	
H4A	0.4611	0.1877	0.3395	0.088*	0.50
H4B	0.6217	0.1899	0.3813	0.088*	0.50
H4C	0.5399	0.3725	0.3600	0.088*	0.50
C5	0.1634 (4)	0.2500	0.6240 (2)	0.0569 (8)	
H5A	0.0870	0.3169	0.5938	0.085*	0.50
H5B	0.1825	0.3054	0.6796	0.085*	0.50
H5C	0.1303	0.1277	0.6327	0.085*	0.50

### Atomic displacement parameters (Å^2^)

**Table:**

	*U*^11^	*U*^22^	*U*^33^	*U*^12^	*U*^13^	*U*^23^
Cl1	0.0355 (4)	0.1078 (8)	0.0747 (6)	0.000	0.0014 (4)	0.000
Cl2	0.0725 (4)	0.0603 (4)	0.0570 (4)	0.0022 (3)	−0.0091 (3)	−0.0172 (3)
Al1	0.0347 (4)	0.0493 (5)	0.0356 (4)	0.000	−0.0053 (3)	0.000
N1	0.0334 (12)	0.0518 (14)	0.0430 (12)	0.000	0.0001 (10)	0.000
N2	0.0395 (12)	0.0463 (13)	0.0357 (11)	0.000	−0.0021 (9)	0.000
C1	0.0334 (13)	0.0405 (14)	0.0348 (12)	0.000	−0.0006 (10)	0.000
C2	0.0343 (15)	0.071 (2)	0.0590 (18)	0.000	−0.0129 (14)	0.000
C3	0.0503 (17)	0.068 (2)	0.0431 (15)	0.000	−0.0162 (14)	0.000
C4	0.0402 (16)	0.084 (2)	0.0524 (17)	0.000	0.0116 (14)	0.000
C5	0.0509 (18)	0.081 (2)	0.0387 (15)	0.000	0.0058 (13)	0.000

### Geometric parameters (Å, º)

**Table:**

Cl1—Al1	2.1193 (12)	C2—C3	1.332 (5)
Cl2—Al1	2.1290 (7)	C2—H2	0.9300
Al1—C1	2.006 (3)	C3—H3	0.9300
Al1—Cl2^i^	2.1291 (7)	C4—H4A	0.9600
N1—C1	1.352 (3)	C4—H4B	0.9600
N1—C2	1.368 (4)	C4—H4C	0.9600
N1—C4	1.468 (4)	C5—H5A	0.9600
N2—C1	1.356 (3)	C5—H5B	0.9600
N2—C3	1.373 (4)	C5—H5C	0.9600
N2—C5	1.460 (4)		
			
C1—Al1—Cl1	113.32 (9)	N2—C1—Al1	131.37 (19)
C1—Al1—Cl2	106.74 (5)	C3—C2—N1	107.2 (3)
Cl1—Al1—Cl2	109.45 (3)	C3—C2—H2	126.4
C1—Al1—Cl2^i^	106.74 (5)	N1—C2—H2	126.4
Cl1—Al1—Cl2^i^	109.45 (3)	C2—C3—N2	107.3 (3)
Cl2—Al1—Cl2^i^	111.11 (5)	C2—C3—H3	126.4
C1—N1—C2	110.7 (2)	N2—C3—H3	126.4
C1—N1—C4	125.3 (2)	N1—C4—H4A	109.5
C2—N1—C4	124.0 (2)	N1—C4—H4B	109.5
C1—N2—C3	110.3 (2)	N1—C4—H4C	109.5
C1—N2—C5	126.4 (2)	N2—C5—H5A	109.5
C3—N2—C5	123.3 (2)	N2—C5—H5B	109.5
N1—C1—N2	104.6 (2)	N2—C5—H5C	109.5
N1—C1—Al1	124.02 (19)		
			
C2—N1—C1—N2	0.0	Cl2^i^—Al1—C1—N1	−59.45 (4)
C4—N1—C1—N2	180.0	Cl1—Al1—C1—N2	0.0
C2—N1—C1—Al1	180.0	Cl2—Al1—C1—N2	−120.55 (4)
C4—N1—C1—Al1	0.0	Cl2^i^—Al1—C1—N2	120.55 (4)
C3—N2—C1—N1	0.0	C1—N1—C2—C3	0.0
C5—N2—C1—N1	180.0	C4—N1—C2—C3	180.0
C3—N2—C1—Al1	180.0	N1—C2—C3—N2	0.0
C5—N2—C1—Al1	0.0	C1—N2—C3—C2	0.0
Cl1—Al1—C1—N1	180.0	C5—N2—C3—C2	180.0
Cl2—Al1—C1—N1	59.45 (4)		

Symmetry code: (i) *x*, −*y*+1/2, *z*.
